# Prevalence and Risk Factors of Human Papillomavirus Infection among Japanese Female People: A Nationwide Epidemiological Survey by Self-Sampling

**DOI:** 10.31557/APJCP.2021.22.6.1843

**Published:** 2021-06

**Authors:** Tadaichi Kitamura, Motofumi Suzuki, Kazuyoshi Shigehara, Kazuko Fukuda

**Affiliations:** 1 *Japanese Foundation for Sexual Health Medicine, Tokyo, Japan. *; 2 *Department of Urology, Tokyo Metropolitan Bokutoh Hospital, Tokyo, Japan. *; 3 *Department of Urology, Faculty of Medicine, Kanazawa University, Kanazawa, Japan. *; 4 *The Postgraduate Course, Sahlgrenska Academy at University of Gothenburg, Gothenburg, Sweden. *

**Keywords:** Human papillomavirus, epidemiology, Japanese women, cross-sectional study, self-sampling

## Abstract

**Background:**

Nationwide epidemiological surveys of behavioral factors and human papillomavirus (HPV) infection among Japanese women are scarce. This study aimed to determine the prevalence, genotype distribution, and significant predictive factors of HPV infection using self-collected vaginal samples from Japanese female people.

**Methods:**

The study population consisted of 1,050 female subjects aged 16–75 years (median 30 years). The participants were asked to provide self-collected samples from the vaginal wall using cotton swabs for genotyping of HPV. We compared the participants’ characteristics and detected HPV genotypes to determine significant predictors of HPV infection.

**Results:**

After excluding 47 participants (34 participants of unknown age, 11 virgin participants, 1 participant who engaged in sex with another woman, and 1 participant who did not undergo β-globin detection), 1,003 participants were included in the analysis. Of the 1,003 participants, 426 (42.5%) participants had at least one HPV genotype, 282 (28.1%) participants had high-risk HPV genotypes, 306 (30.5%) had low-risk HPV genotypes, and 162 (16.2%) participants had both HPV genotypes. HPV-16/18 positivity was found in 5.4% (54/1,003) participants. The most frequently detected high-risk HPV genotype was HPV-52 (86/1,003; 8.6% participants). The number of lifetime sex partners (≥6) and a present history of sexually transmitted infection (STI) were significant predictors of high-risk HPV infection. The number of lifetime sex partners (≥6), age of coitarche (≥20 years of age), unmarried status, and a present history of STI were significant predictors of low-risk HPV infection.

**Conclusions:**

The prevalence of high-risk and low-risk HPV infection among Japanese female subjects was 28.1% and 30.5%, respectively. The number of lifetime sex partners (≥6) and present history of sexually transmitted infection were the common significant predictors of high-risk and low-risk HPV infection.

## Introduction

In 2018, approximately 570,000 women were diagnosed with cervical cancer worldwide and 311,000 women died of this disease. Cervical cancer is the fourth most common cancer in women, ranking after breast cancer, colorectal cancer, and lung cancer (Arbyn et al., 2020). According to the recent statistics (Ferlay et al., 2018), the estimated age-standardized incidence rate of cervical cancer in Japan is 14.7/100,000 person-years; however, it is far from elimination of cervical cancer (<4.0/100,000 person-years). Moreover, the estimated age-standardized mortality rate of cervical cancer in Japan is 2.7/100,000 person-years.

Since almost all cervical cancer cases are due to infection with high-risk human papillomavirus (HPV) transmitted through sexual intercourse, primary (HPV vaccination) and secondary prevention approaches (screening and treating precancerous lesions of the uterine cervix) are effective to prevent cervical cancer (Arbyn et al., 2020). In Japan, the bivalent HPV vaccine became available in October 2009, followed by the quadrivalent HPV vaccine in August 2011. The nonavalent HPV vaccine was approved by the Ministry of Health, Labour and Welfare of Japan in July 2020; however, it has not been distributed in Japan thus far; this vaccine needs to be imported individually for use. Funding for HPV vaccination in Japan began in 2010 for girls aged 12–16 years, with three-dose coverage initially reaching >70%. However, due to the occurrence of various symptoms including chronic pain and motor impairment, the Ministry of Health, Labour and Welfare of Japan announced the suspension of the proactive recommendation for the routine use of HPV vaccine in the national immunization program in June 2013, resulting in a vaccine coverage rate of <1% (Hanley et al., 2015). Hence, the World Health Organization’s Global Advisory Committee on Vaccine Safety commented that young Japanese women have been left vulnerable to preventable HPV-related cancers (Ikeda et al., 2021). Recently, Simms (2020) reported that the vaccine crisis resulted in an estimated 5,000 deaths from cervical cancer in Japan.

Not only vaccination prior to coitarche but also preventive measures including consistent and correct use of latex condom and having a mutually monogamous relationship are important strategies to prevent HPV infection (Hamdi, 2018). Regarding the risk factors of HPV infection, Pourmohsen (2018) reviewed 29 articles on the HPV infection risk factors and preventive behaviors. Individual factors (ethnicity, age, education, social class and income, occupation, marital status, and parity) and behavioral factors (smoking, age of marriage or first intercourse, and monogamy or polygamy) were listed; however, most of these factors were controversial due to lack of consensus among the researchers. Depending on the different context of society, culture, and economy, there are many confounding factors of HPV infection. Meanwhile, number of pregnancies/parity, multiplicity of sex partners as well as polygamy, and smoking are common risk factors of HPV infection.

From a clinical point of view, it is important to conduct epidemiological studies on HPV infection among women in each country to identify their own predictors of HPV infection. Based on these studies, behavioral modifications can be suggested to help reduce the prevalence of HPV infection among women. However, limited epidemiological studies on HPV infection have conducted among Japanese female people. Hence, we conducted a nationwide, cross-sectional study to determine the prevalence, genotype distribution, and predictors of HPV infection among Japanese female people.

Regarding the reliability of self-collected vaginal sample; unfortunately, HPV testing using self-sampling has not been recommended in Japan because it is unclear whether it will increase the gynecological clinic visits. However, we had previously reported that HPV DNA positivity was 78.4% (58/74) in self-collected samples and 70.3% (52/74) in gynecologist-collected samples (P=0.26) (Kitamura et al., 2017). Moreover, a combination of HPV testing via self-sampling and a follow-up Pap test is more sensitive than a Pap test alone for the detection of HPV (Gupta, 2018). Recently, HPV testing using self-collected vaginal samples has been implemented in the Netherlands, Australia, the United States, the United Kingdom, Norway, Denmark, and Switzerland (El-Zein, 2019). Hence, we selected HPV testing via self-sampling in the present study.

## Materials and Methods

From April 2017 to March 2020, we conducted a nationwide, cross-sectional study to determine the prevalence, genotype distribution, and predictors of HPV infection among Japanese female people. Japanese female subjects were recruited via the Japanese Foundation for Sexual Health Medicine website (https://www.jfshm.org/). This website can be accessed all through the world; however, participants had accessed domestically because all the contents were written in Japanese language. When a female suject agreed to participate in the survey through our homepage, we sent her a cotton swab with a plastic tube container and a printed survey form to collect information on age, educational attainment, smoking status, number of lifetime sex partners, age of coitarche, present marital status, experience of divorce, number of children, commercial sex work experience, present history of sexually transmitted infection (STI), past history of STI, and HPV vaccination status. Except during the menstrual period, the participants were asked to self-collect a vaginal sample by rubbing a cotton swab on the vaginal wall for several times and place the sample in a plastic tube container. Subsequently, the participants were asked to send the sample to our office and complete the survey form.

In this study, self-collected vaginal samples were shipped to LSI Medience Corporation (Tokyo, Japan), and types of HPV DNA and β-globin were detected using the GENOSEARCH HPV31 kit (Medical and Biological Laboratory, Nagoya, Japan) according to the manufacturer’ s instructions (Imai et al., 2015). Briefly, this kit employs polymerase chain reaction with the reverse sequence-specific oligonucleotide probe method to detect HPV DNA. The test kit can detect the following 31 types of HPV DNA: high-risk types (types 16, 18, 26, 31, 33, 35, 39, 45, 51, 52, 53, 56, 58, 59, 66, 68, 70, 73, and 82) and low-risk types (types 6b, 11, 42, 44, 54, 55, 61, 62, 71, 84, 90, and CP6108) (Bouvard et al., 2009). It also contains a probe for detecting human β-globin to ensure that self-collected vaginal samples contain sufficient cellular components. All protocols and assessments used in this study were approved by the ethics committees of the Japanese Foundation for Sexual Health Medicine (JFSHM No. 1 and JFSHM No. 5). Written informed consent was obtained from all participants.

Statistical analyses were performed using JMP Pro software version 15.0.0 (SAS, Cary, NC). The chi-square test was used to compare the prevalence of HPV infection among the age groups (10-year stratification). Predictors of HPV infection were analyzed using logistic regression analysis. In multivariate analysis, all covariates were included for the risk calculation. P-values <0.05 were considered statistically significant.

## Results

The study population consisted of 1,050 Japanese female citizens. After excluding 47 participants (34 participants with unknown age, 11 virgin participants, 1 participant who engaged in sex with another woman, and 1 participant who did not undergo β-globin detection), 1,003 participants were included in the analysis. The characteristics of the study participants are shown in Table 1. In total, 8.8% (87/992) participants had a commercial sex work experience—8.7% participants were aged 16–19 years group, 9.6% participants were in their 20s, 10.2% participants were in their 30s, 6.9% participants were in their 40s, and 0% participants were aged ≥50 years, respectively (P=0.021). Although the types of HPV vaccine (bivalent or quadrivalent) were not recorded, only 19.3% (192/994) participants were vaccinated with HPV. Of the 1,003 participants, 426 (42.5%) participants had at least one HPV genotype, 282 (28.1%) participants had high-risk HPV genotypes, 306 (30.5%) participants had low-risk HPV genotypes, and 162 (16.2%) participants had both genotypes. HPV-16/18 positivity was reported in 5.4% (54/1,003) participants. The most frequently detected high-risk HPV genotype was HPV-52 (86/1,003; 8.6% participants; Table 2). The prevalence of high-risk HPV genotypes was 20.8% in participants aged 16–19 years, 34.0% in participants in their 20s, 25.7% in participants in their 30s, 22.4% in participants in their 40s, and 13.2% in participants aged ≥50 years (P=0.0009). The prevalence of low-risk HPV genotypes was 29.2% in participants aged 16–19 years, 36.8% in participants in their 20s, 26.0% in participants in their 30s, 23.6% in participants in their 40s, and 24.5% in participants aged ≥50 years (P=0.0026).

Logistic regression analyses revealed that the number of lifetime sex partners (≥6) and a present history of STI were predictors of high-risk HPV infection. Commercial sex work experience was significantly associated with high-risk HPV infection in univariate analysis, but not in multivariate analysis (Table 3). Conversely, the number of lifetime sex partners (≥6), age of coitarche (≥20 years of age), unmarried status, and a present history of STI were predictors of low-risk HPV infection (Table 4). HPV vaccination was not associated with either high-risk or low-risk HPV infection.

**Table 1:** Characteristics of Japanese Female Ppopulation Participated in the Study

Parameters	All (n=1,003)
Age, years	
Median (range)	30 (16–75)
Age group, n (%)	
16–19 years	24 (2.4%)
20–29 years	456 (45.5%)
30–39 years	296 (29.5%)
40–49 years	174 (17.3%)
≥50 years	53 (5.3%)
Educational attainment, n (%)	
≤12 years	167 (16.8%)
>12 years	828 (83.2%)
Unknown	8
Smoking status, n (%)	
Never	738 (74.3%)
Current	92 (9.3%)
Former	163 (16.4%)
Unknown	10
Number of lifetime sex partners, n (%)	
1–5	500 (51.5%)
6–10	199 (20.5%)
11–20	143 (14.7%)
≥21	128 (13.2%)
Unknown	33
Age of coitarche, n (%)	
≤19 years	566 (59.6%)
≥20 years	383 (40.4%)
Unknown	54
Present marital status, n (%)	
Married	341 (34.3%)
Not Married	653 (65.7%)
Unknown	9
Divorce, n (%)	
Never	902 (91.0%)
Experienced	89 (9.0%)
Unknown	12
Number of children	
0	708 (71.2%)
1	102 (10.3%)
2	132 (13.3%)
≥3	53 (5.3%)
Unknown	8
Commercial sex work experience, n (%)	
No	905 (91.2%)
Yes	87 (8.8%)
Unknown	11
Present history of STI, n (%)	
No	945 (95.4%)
Yes	46 (4.6%)
Parameters	All (n=1,003)
Present history of STI, n (%)	
Unknown	12
Past history of STI, n (%)	
No	717 (72.4%)
Yes	274 (27.6%)
Unknown	12
HPV vaccination status, n (%)	
No	802 (80.7%)
Yes	192 (19.3%)
Unknown	9

STI, sexually transmitted infection; HPV, human papillomavirus.

**Table 2:** Number of HPV Positive Cases among 1,003 Japanese Female Participants

	Number of HPV positive cases, n (%)
Types of HPV	
Any type	426 (42.5%)
High-risk	282 (28.1%)
Low-risk	306 (30.5%)
Both	162 (16.2%)
HPV-16/18	54 (5.4%)
High-risk HPV genotypes	
HPV-16	38 (3.8%)
HPV-18	17 (1.7%)
HPV-26	2 (0.2%)
HPV-31	14 (1.4%)
HPV-33	12 (1.2%)
HPV-35	8 (0.8%)
HPV-39	38 (3.8%)
HPV-45	11 (1.1%)
HPV-51	46 (4.6%)
HPV-52	86 (8.6%)
HPV-53	49 (4.9%)
HPV-56	47 (4.7%)
HPV-58	43 (4.3%)
HPV-59	26 (2.6%)
HPV-66	38 (3.8%)
HPV-68	34 (3.4%)
HPV-70	3 (0.3%)
HPV-73	12 (1.2%)
HPV-82	25 (2.5%)
Low-risk HPV genotypes	
HPV-6b	50 (5.0%)
HPV-11	8 (0.8%)
HPV-42	36 (3.6%)
HPV-44	7 (0.7%)
HPV-54	25 (2.5%)
HPV-55	36 (3.6%)
HPV-61	44 (4.4%)
HPV-62	59 (5.9%)
HPV-71	18 (1.8%)
HPV-84	47 (4.7%)
HPV-90	63 (6.3%)
HPV-CP6108	32 (3.2%)

HPV, human papillomavirus.

**Table 3:** Predictors of high-risk HPV Infection among Japanese Women

	Crude OR OR (95% CI)	Adjusted OR* OR (95% CI)
Age group (years)		
16–19	Reference	Reference
20–29	1.957 (0.717–5.340)	2.406 (0.659–8.793)
30–39	1.313 (0.474–3.637)	1.464 (0.388–5.530)
40–49	1.098 (0.385–3.129)	1.264 (0.311–5.141)
≥50	0.578 (0.163–2.051)	1.859 (0.349–9.891)
Educational attainment		
≤12 years	Reference	Reference
>12 years	0.835 (0.582–1.199)	1.178 (0.733–1.894)
Smoking status		
Never	Reference	Reference
Former	1.725 (1.201–2.478)	1.477 (0.942–2.316)
Current	2.741 (1.760–4.267)	1.218 (0.708–2.095)
Number of lifetime sex partners		
1–5	Reference	Reference
6–10	2.784 (1.857–4.176)	2.752 (1.753–4.321)
11–20	7.439 (4.882–11.335)	7.428 (4.467–12.353)
≥21+	10.821 (6.951–16.845)	11.873 (6.537–21.567)
Age of coitarche		
≤19	Reference	Reference
≥20	0.543 (0.402–0.734)	1.048 (0.715–1.537)
Present marital status		
Married	Reference	Reference
Unmarried	2.255 (1.638–3.105)	1.379 (0.814–2.335)
Divorce		
Never	Reference	Reference
Experienced	1.425 (0.900–2.258)	1.243 (0.658–2.347)
Number of children		
0	Reference	Reference
1	0.520 (0.311–0.869)	0.624 (0.290–1.342)
2	0.548 (0.349–0.861)	0.919 (0.446–1.895)
≥3	0.325 (0.144–0.730)	0.502 (0.178–1.415)
Commercial sex work experience		
No	Reference	Reference
Yes	2.512 (1.608–3.925)	0.671 (0.373–1.205)
Present history of STI		
No	Reference	Reference
Yes	4.324 (2.351–7.955)	2.891 (1.383–6.043)
Past history of STI		
No	Reference	Reference
Yes	2.458 (1.827–3.308)	1.180 (0.792–1.758)
HPV vaccination status		
No	Reference	Reference
Yes	1.124 (0.796–1.586)	0.979 (0.629–1.526)

OR, odds ratio; CI, confidence interval; STI, sexually transmitted infection; HPV, human papillomavirus; *, Multivariate analysis was performed including all variables.

**Table 4:** Predictors of Low-Risk HPV Infection among Japanese Women

	Crude OR OR (95% CI)	Adjusted OR* OR (95% CI)
Age group (years)		
16–19	Reference	Reference
20–29	1.417 (0.576–3.486)	0.906 (0.290–2.828)
30–39	0.854 (0.341–2.138)	0.676 (0.209–2.187)
40–49	0.749 (0.290–1.931)	0.724 (0.208–2.523)
≥50	0.789 (0.268–2.324)	1.582 (0.367–6.817)
Educational attainment		
≤12 years	Reference	Reference
>12 years	0.774 (0.545–1.099)	0.900 (0.572–1.416)
Smoking status		
Never	Reference	Reference
Former	1.172 (0.814–1.687)	0.913 (0.583–1.431)
Current	1.564 (0.999–2.447)	0.685 (0.397–1.183)
Number of lifetime sex partners		
1–5	Reference	Reference
6–10	3.143 (2.150–4.596)	3.649 (2.374–5.610)
11–20	5.746 (3.815–8.655)	8.442 (5.073–14.049)
≥21	9.136 (5.927–14.083)	13.033 (7.161–23.718)
Age of coitarche		
≤19	Reference	Reference
≥20	0.765 (0.577–1.015)	1.635 (1.125–2.375)
Present marital status		
Married	Reference	Reference
Unmarried	2.564 (1.869–3.519)	1.780 (1.061–2.985)
Divorce		
Never	Reference	Reference
Experienced	1.178 (0.742–1.869)	0.795 (0.424–1.490)
Number of children		
0	Reference	Reference
1	0.547 (0.335–0.892)	1.192 (0.592–2.397)
2	0.376 (0.232–0.609)	0.694 (0.334–1.442)
≥3	0.550 (0.284–1.065)	0.744 (0.290–1.912)
Commercial sex work experience		
No	Reference	Reference
Yes	2.438 (1.563–3.801)	0.668 (0.375–1.191)
Present history of STI		
No	Reference	Reference
Yes	5.655 (2.971–10.765)	3.664 (1.703–7.882)
Past history of STI		
No	Reference	Reference
Yes	2.412 (1.802–3.229)	1.223 (0.830–1.802)
HPV vaccination status		
No	Reference	Reference
Yes	1.424 (1.024–1.982)	1.325 (0.868–2.022)

OR, odds ratio; CI, confidence interval; STI, sexually transmitted infection; HPV, human papillomavirus; *, Multivariate analysis was performed including all variables.

## Discussion

Epidemiological surveys of HPV infection among Japanese women are scarce. Two epidemiological studies have been conducted in the northern area of mainland Japan. Sasagawa (2005) surveyed 781 Japanese women who attended gynecological clinics and reported that the prevalence of high-risk HPV infection was 40% in women aged 15–24 years, 19% in women aged 25–34 years, 11% in women aged 35–34 years, and 16% women aged in 45–59 years. Predictors of high-risk HPV infection were single status, a history of STI, the notion of having an STI, and increased numbers of sex partners. The study also reported that the prevalence of high-risk HPV infection was 50% in women aged 15–19 years and 37% in women aged 20–24 years. These prevalence rates were very similar to those reported among Japanese commercial sex workers (48.4%; mean age, 29 years) (Ishii et al., 2000). Kurokawa (2018) conducted a large-scale epidemiological survey with 7,585 Japanese female participants who accepted the cervical cancer screening program. The prevalence of high-risk HPV infection was 16.5% in participants aged 25–29 years, 10.1% in participants in their 30s, 5.6% in participants in their 40s, and 3.0% in participants aged ≥50 years. The present nationwide survey included 1,003 Japanese female participants who applied for our proposal. The prevalence of high-risk HPV genotypes was 20.8% in participants aged 16–19 years, 34.0% in participants in their 20s, 25.7% in participants in their 30s, 22.4% in participants in their 40s, and 13.2% in participants aged ≥50 years (P=0.0009) and that of low-risk HPV genotypes was 29.2% in participants aged 16–19 years, 36.8% in participants in their 20s, 26.0% in participants in their 30s, 23.6% in participants in their 40s, and 24.5% in participants aged ≥50 years (P=0.0026). The prevalence of HPV infection among participants in their 20s in our study was similar to that reported by Sasagawa et al., (2005). Although the actual proportion of Japanese female participants who had commercial sex work experience is unknown, since our cohort included women who had experience of commercial sex work (8.8%), they might be more interested in HPV infection than the cohort recruited by Kurokawa et al., (2018). Our study revealed that the number of lifetime sex partners (≥6) and a present history of STI were significant predictors of high-risk HPV infection, but not present marital status, as suggested by Sasagawa et al., (2005). In a study including a southern Chinese female cohort, Liu et al., (2011) suggested that age was an independent risk factor for HPV infection in the Hong Kong cohort, while the number of sexual partners was an independent risk factor for HPV infection in the Guangzhou cohort. Risk factors for HPV infection may vary depending on the area of residence. Kum-Nji et al., (2019) reported that both passive and active tobacco smoking were strongly associated with HPV infection in 1,414 young women in the United States aged 18–26 years, with an odds ratio (OR) (95% confidence interval [CI]) of 2.45 (1.34–4.48) and 3.56 (1.23–10.30), respectively. Torres-Poveda et al., (2019) reported that tobacco smoking was significantly associated with high-risk HPV infections, except for HPV-18 in 15,040 Mexican women aged 18–89 years. In the Japanese epidemiological survey, current tobacco smoking was not associated with HPV infection (Sasagawa et al., 2005). Even in Japanese male subjects, a history of current or past tobacco smoking was not associated with HPV infection (Matsuzawa et al., 2020). We are interested in whether male subjects living in the same province or area harbor the similar risk factors for HPV infection as the female subjects.

High-risk HPV infection is associated with not only cervical cancer but also female infertility. In a meta-analysis, Yuan (2019) reported that high-risk HPV infection was significantly associated with female infertility with an OR (95% CI) of 2.33 (1.42–3.83). Hsu (2020) reported the relationship between a history of HPV infection and female infertility in a female cohort. Although their study did not account for HPV genotypes, the female cohort with a history of HPV infection had an increased risk of infertility, with a hazard ratio (95% CI) of 1.39 (1.19–1.63). To the best of our knowledge, there is no epidemiological study on the association between HPV infection and female/male infertility among Japanese subjects.

In a Swedish female study, Lei et al., (2020) reported that quadrivalent HPV vaccination was associated with a substantially reduced risk of invasive cervical cancer with an incidence rate (95% CI) of 0.12 (0.00–0.34) among women who had been vaccinated before the age of 17 years and 0.47 (0.27–0.75) among women who had been vaccinated between the age of 17 and 30 years. Even in Japan, the effectiveness of HPV vaccination has been clarified. Ikeda et al., (2020) reported that the OR (95% CI) of HPV vaccination compared with no vaccination for abnormal cytology, cervical intraepithelial neoplasia (CIN)1+, CIN2+, and CIN3+ versus control was 0.42 (0.34–0.50), 0.42 (0.31–0.58), 0.25 (0.12–0.54), and 0.19 (0.03–1.15), respectively, equating to a vaccine effectiveness of 58.5%, 57.9%, 74.8%, and 80.9%, respectively. It is unclear which HPV vaccines (bivalent, quadrivalent, and nonavalent) can effectively reduce HPV-related diseases (carcinoma of the cervix, vulva, vagina, penis, anus, oral cavity, oropharynx, and tonsil; genital warts; and recurrent respiratory papillomatosis).

The present study has the following limitations. Our cohort included not a general population but female participants who applied to our proposal published on our homepage. The participants might have a higher health literacy than the general female population. We also did not have data on the type of HPV vaccine applied to the participants (bivalent or quadrivalent) or their age of ingestion. Although our study had such limitations, we would like to emphasize that the dissemination of self-sampling of the vaginal wall to detect high-risk HPVs is important for promoting health awareness and directing infected women to gynecological clinics in Japan. Detecting HPV-6 and HPV-11 is also important for protecting future babies from developing recurrent respiratory papillomatosis. We understand that our campaign (https://www.jfshm.org/doc/H30_HPVtest.pdf?c=201804090001) is only an alternative to reduce the development of HPV infections among unvaccinated female people. However, we recommend gynecological examinations for Japanese female high-risk HPV carriers to detect cervical cancer throughout our campaign just under the current policy of prolonged suspension of recommendation of HPV vaccination by the Ministry of Health, Labour and Welfare of Japan.

Our study included 87 (8.8%) women who had commercial sex work experience; however, commercial sex work experience was not found to be a predictor for high-risk or low-risk HPV infection. The number of lifetime sex partners (≥6) and a present history of STI were a reported to be significant risk factor for both high-risk and low-risk HPV infection. Behavioral modifications, including a lower number of lifetime sex partners and protection against STI (e.g., condom use) might be crucial to prevent HPV infections until the Ministry of Health, Labour and Welfare of Japan resumes the proactive recommendation for the routine use of the HPV vaccine in the national immunization program.

## Author Contribution Statement

Tadaichi Kitamura designed the study and wrote the initial draft of the manuscript. Motofumi Suzuki performed statistical analysis of the data and wrote the initial draft of the manuscript. Kazuyoshi Shigehara and Kazuko Fukuda contributed to interpretation of data and critically reviewed the manuscript. All authors approved the final version of the manuscript and agree to be accountable for all aspects of the work in ensuring that questions related to the accuracy or integrity of any part of the work are appropriately investigated and resolved.
